# Classical Hodgkin's lymphoma with cutaneous involvement in an adolescent male: A case study

**DOI:** 10.1002/cnr2.1473

**Published:** 2021-06-05

**Authors:** Sumon Ghosh, Sajib Ghosh, Rownak Jahan Amin, Fahmida Chowdhury, Namala Satya Prasad, Pandurangan Prabu, Sukanta Chowdhury

**Affiliations:** ^1^ Infectious Diseases Division International Centre for Diarrhoeal Disease Research, Bangladesh (icddr,b) Dhaka Bangladesh; ^2^ Department of Pathology Eastern Medical College & Hospital Cumilla Bangladesh; ^3^ Department of Radiation Oncology National Institute of Cancer Research and Hospital Dhaka Bangladesh; ^4^ Department of Haematology Apollo Hospital Chennai India

**Keywords:** ABVD, case report, chemotherapy, cutaneous lesions, Hodgkin lymphoma, lymphoma

## Abstract

**Background:**

Hodgkin's lymphoma (HL) with skin involvement is reasonably rare. It typically occurs late in the course and is associated with a poor prognosis; however, it may also be indolent in some cases.

**Case:**

We report a case of a 15‐year‐old previously healthy male with Hodgkin's lymphoma who presented with multiple lymphadenopathies of axilla and serpiginous ulcerative nodular lesions involving pectoral skin. A lymph node biopsy was performed following an initial diagnostic workup for a suspected active infectious disease, which revealed a neoplastic invasion from a mixed cellularity classical HL with skin involvement. A total of six cycles of ABVD (doxorubicin, bleomycin, vinblastine, and dacarbazine) chemotherapy regimen was administered to the patient.

**Conclusion:**

In comparison to other studies, this case demonstrates that a good response is possible with standard ABVD chemotherapy.

## 1 INTRODUCTION

Hodgkin's lymphoma (HL), previously referred to as Hodgkin's disease, is a type of lymphoma triggered by lymphocytes and accounts for 0.58% of all diagnosed cancers.^1^, ^2^ It starts in lymph nodes and spread from one group of lymph nodes to another.^1^ It is characterized by nodal infiltration, typically involving cervical, mediastinal, and axillary regions (accounting for almost 90% of HL presentations), while extranodal involvement occurs in almost a quarter of cases, more often secondary to contiguous spreading from bulky masses.^3^ Patients typically have painless lymphadenopathy. B symptoms, such as unexplained fever, drenching night sweats, and weight loss, occur in one‐third of the patients.^4^

Involvement of skin in HL is rare and seen in only 0.5%–3.5% of cases.^5^ Skin involvement can occur through various routes, including retrograde lymphatic spread, direct extension from underlying nodes, or hematogenous spread. The prognosis of HL is usually determined by the degree of visceral involvement and the stage of the disease.^6^ Cutaneous HL is generally seen in stage IV disease and is associated with a dismal prognosis; however, if the systemic disease is well controlled, it might follow an indolent course.^7^

We report a case of a 15‐year‐old male patient with HL who presented initially with fever, multiple cervical and axillary lymphadenopathy, and bilateral cutaneous pectoral lesions.

## 2 CASE

### 2.1 Case presentation

A 15‐year‐old male patient, born in Bangladesh, was admitted to our facility in March 2019 with complaints of the swelling chest wall for 3 months and painless multiple cutaneous nodules with a history of watery discharge in the anterior chest wall, which had progressively increased in size. This was associated with an on‐and‐off fever, night sweats, and anorexia with more than a 6‐kg loss of weight over the previous 3 months. The patient also had bilateral cervical and axillary lymphadenopathy, showing no signs of improvement after a broad‐spectrum antibiotics treatment prescribed by the general practitioner. He also took anti‐tubercular treatment for approximately 2 months but failed to find any relief. It is important to note that there was no significant family history of a similar disease.

### 2.2 Physical examination upon admission

**FIGURE 1:**
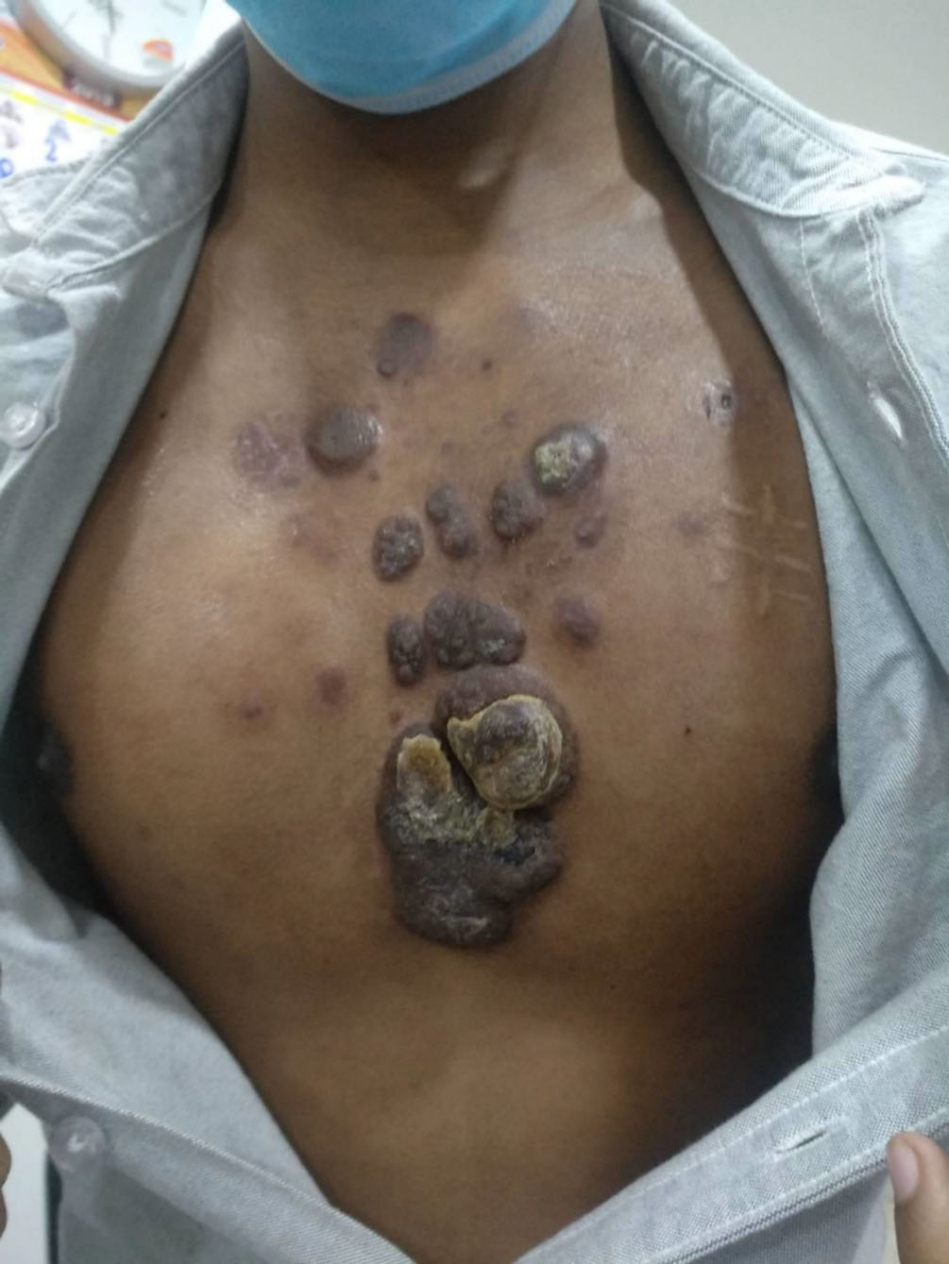
Pre‐chemotherapy photograph showing nodular mass with ulceration over the mid‐chest region

Physical examination revealed afebrile condition with multiple erythematous and serpiginous nodular lesions varying in size from 1 × 1 cm^2^ to 6 × 5 cm^2^ on the anterior chest wall, protruding right mammary region, collection intercostals space (Figure 1). Bilateral axillary lymphadenopathy in the left posterior triangle was also observed.

### 2.3 Laboratory examinations

Laboratory data reported an increased white blood count (23 860/mm^3^) with neutrophilia (77%), platelets (612 000/mm^3^), C‐reactive protein (CRP; 171 mg/L), and erythrocyte sedimentation rate (ESR; 39 mm). The viral screen was negative for human immunodeficiency virus (HIV) and hepatitis B surface antigen (HBsAg). No acid‐fast bacillus (AFB) was seen on AFB testing (Table 1). Bone marrow aspiration cytology (BMA) showed normocellular marrow with trilineage hematopoiesis, a mild increase in eosinophils, and plasma cells (Table 2). Bone marrow (trephine) biopsy showed normocellular marrow with mild eosinophilia and plasmacytosis with negative focal lesions (Table 3).

**TABLE 1:** Biochemical and hematological features of the samples collected from the patient before chemotherapy

Specimen collection date	Specimen	Test name	Result	Biological reference intervals	Units
March 12, 2019	Serum	Urea ‐ Serum/Plasma	20	Adult: 13–43	mg/dl
		AST (SGOT) ‐ Serum	15	10–19 years: 5–45	U/L
March 12, 2019	Whole blood (EDTA)	CBC			
		Hemoglobin (Optical[light scatter]/Cyanmethhaemoglobin)	11.3^a^	13–16	gm%
		Packed cell volume (Calculated)	36^a^	37–49	%
		WBC count (Optical/Impedance)	23.86^a^	4.5–13.5	10^3^/mm^3^
		Platelet Count (Optical [light scatter]/Derived from platelet histogram)	612^a^	150–450	10^3^/mm^3^
		ESR (Automated ‐ Westergren method)	39^a^	0–15	mm/hr
March 12, 2019	Whole blood (EDTA)	Differential Count (Optical[light scatter]/VCS/Microscopy)			
		Neutrophils	77^a^	33–76	%
		Lymphocytes	18	15–55	%
		Eosinophils	1	0–3	%
		Monocytes	4	0–4	%
March 12, 2019	Whole blood (EDTA)	Red cell morphology (Microscopy)			
		Target cells	Occasional		
		RBC	Microcytic Hypochromic RBCs		
		WBC	Leucocytosis noted		
		Platelets	Increased on smear		
March 13, 2019	Pus (Chest nodules)	Culture and Sensitivity [PUS]: (Culture)			
		Gram stain	Few pus cells and occasional Gram positive cocci in pairs seen		
		Organism isolated:	Scanty growth of *Staphylococcus haemolyticus*		
		Identification method	MALDI‐TOF (Vitek MS)		
		Growth observed	Insignificant		
March 15, 2019	Blood	C‐reactive protein (CRP) (Nephelometry)	171^a^	Normal: <5.0	mg/L
March 15, 2019	Blood	HBsAg	CMIA		
		Methodology	Negative		
		AIDS/HIV	CMIA		
		Methodology	Negative		
March 15, 2019	Citrated plasma	Activated partial thromboplastin time: (Light scatter detection)			
		Test:	35^a^	21–33 s	s
		Control	27		s
March 15, 2019	Citrated plasma	Thrombin time: (Light scatter detection)			
		Test:	16		s
		Control	17		
		Prothrombin time: (Light scatter detection)			
		Test:	15^a^	10–13 s	s
		Control	11		s
March 16, 2019	Tissue (chest nodules)	Fungal stain [tissue]: (Conventional) Smear:	No fungal elements seen		
		Gram stain [tissue]: (Conventional)	Few pus cells, occasional epithelial cells and no bacteria seen		
		Culture and sensitivity [tissue]: (Culture)	No growth so far		
		Acid fast stain [tissue] AFB (Acid‐fast bacteria) Smear	No AFB seen		

^a^ Indicates higher value than ref.

**TABLE 2:** Bone marrow haematology before chemotherapy

Specimen collection date	Specimen	Test	Result	Biological reference intervals	Units	Comments	Impression
March 22, 2019	BMA	Bone marrow aspiration cytology [BMA]: (Microscopy)				Bone marrow aspirate smears show normocellular particles and trails. Erythropoiesis is normoblastic in maturation. Myelopoiesis shows progressive maturation sequences. There is increase ineosinophils. Lymphocytes are in the normal range. There is plasmacytosis. Megakaryocytes are mildly increased and normalmorphology. No parasites or granulomas are seen in the smears studied. Iron stores ‐(4+) Peripheral smear shows normocytic hypochromic RBCs with occasional target cell. Leucocytosis seen. There is increase in platelets.	Normocellular marrow with trilineage heamatopoiesis, mild increase in eosinophils and plasma cells
		Myeloblasts	3	−3.5	%		
		Myelocytes	3*	5–20	%		
		Metamyelocytes	9*	10–30	%		
		Neutrophils	37*	7–25	%		
		Eosinophils	7*	−3.0	%		
		Lymphocytes	12	5–20	%		
		Monocytes	4*	0–0.2	%		
		Plasma calls	5*	−3.5	%		
		Normoblasts	20	4–30	%		
		M/E ratio	3.1:1				

* Indicates higher value than ref.

**TABLE 3:** Bone marrow histopathology before chemotherapy

Specimen collection date	Specimen	Test	Macroscopic description	Microscopic description	Impression	Comments
March 22, 2019	Bone marrow (trephine) biopsy	NEEDLE BIOPSY ‐ BONE MARROW	Received single cylindrical greyish brown bony fragment measuring 1.2 cm ‐2all	Section shows multiple linear cores of needle core tissue composed of anastomosing cancellous bony spicules interspersed with marrow adipose tissue and haematopoietic elements. The marrow is normocellular for age with trilineagehaematopoiesis. Erythroid lineage shows normoblastic maturation. Myeloid lineage shows progressive sequential maturation with eosinophilia. Theinterstitiumshows mildplasmacytosis. Megakaryocytes are adequate and appear normal in morphology. Reticulin stain shows no increase in reticulin fibers. Iron stain shows moderately increased iron stores.	Bone marrow (trephine) biopsy showing normocellular marrow with mild eosinophilia and plasmacytosis,negative for focal lesions	Please correlate with bone marrow aspiration cytology findings

### 2.4 Imaging examinations

A whole‐body CT scan revealed prominent bilateral level II and V nodes in head and neck regions. Multiple enlarged left supraclavicular, bilateral axillary, subpectoral, deep pectoral, bilateral internal mammary, and prevascular nodes in the chest were observed where the largest left internal mammary nodal mass extending into the left chest wall (6.0 × 5.5 cm^2^) and left axillary node (3.2 × 3.0 cm^2^). Multiple ill‐defined soft tissue deposits were seen in the anterior chest wall muscles, and necroses were noted within these deposits. Multiple well‐defined cutaneous soft tissue deposits were also seen in the chest wall, and the largest measure was 4.0 × 2.5 cm^2^. Ulceration was noted within a few of the lesions. Nodular pleural thickening was also observed in the bilateral upper hemithorax. No evidence of pleural effusion was found.

**FIGURE 2:**
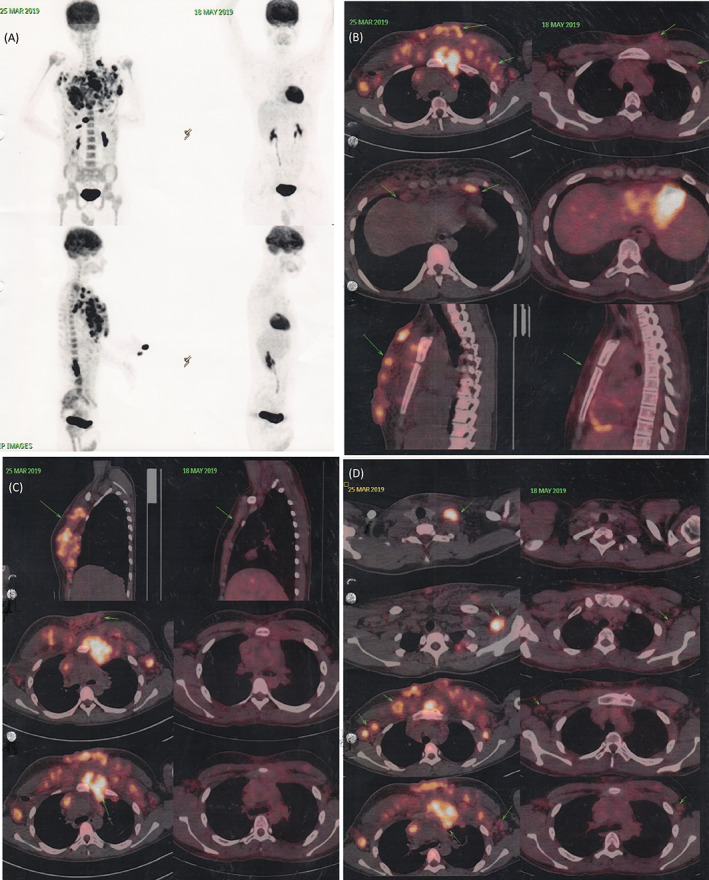
Comparative positron emission tomography‐computed tomography (PET‐CT) and CT imaging before and after chemotherapy (imaging features consistent with lymphoma with coexisting fungal skin infection). (A) A whole body scan showing viable primary pathology involving the lymph node (left) and PET scan done after 3 cycle of ABVD (right), (B–D) Multiple enlarged left supraclavicular, bilateral axillary, subpectoral, deep pectoral, bilateral internal mammary, and prevascular nodes are seen (left) and CT scan done after 2 cycle of ABVD shows significant regression in the previously seen enlarged left supraclavicular, bilateral axillary, subpectoral, deep pectoral, bilateral internal mammary, and perivascular nodes are seen (right). Also significant regression in the multiple ill‐defined soft tissue and subcutaneous, cutaneous deposits are seen in the anterior chest wall muscles are seen (right)

Positron emission tomography (PET) scan revealed increased 18‐fluorodeoxyglucose (FDG) uptake involving enlarged left supraclavicular node (SUV max 10.4), axillary/subpectoral (SUV max right 8.8; left 10.6), prevascular (SUV max 8.6), paraaortic (SUV max 7.1) anterior cardiophrenic (SUV max 9.1), and supradiaphragmatic (SUV max 8.1) nodes, large pleural based deposit in the left internal mammary region (SUV max 11.5), pleural based deposits in both hemithorax (SUV max 9.3), bilateral pectoralis muscle (SUV max right 10.4; left 7.7), and cutaneous and subcutaneous deposits in the anterior chest wall (SUV max 9.3) (Figure 2). Imaging features consistent with lymphoma with a coexisting fungal skin infection.

### 2.5 Final diagnosis

Histopathological examination of the right axillary lymph node and nodular lesions on the chest wall by excision biopsy got the impression of atypical lymphoid infiltrates, consistent with lymphoma (Table 4). Immunohistochemistry (Lymphoma panel) of right axillary lymph node and “nodular lesion,” chest wall, excision biopsy got the impression of consistent with classical Hodgkin Lymphoma, nodular sclerosis with stage 2B disease (Table 5). Lymph node biopsy revealed an immunohistochemical staining positive for CD15, CD30, PAX5, and negative for CD3 and CD20. Nodal tissue was also examined for microbiological testing, which excluded the presence of active tubercular infection.

**TABLE 4:** Histopathological features of specimen collected from the patient before chemotherapy

Specimen collection date	Specimen	Macroscopic lesions	Microscopic lesions	Impression	Comments
March 16, 2019	1. Right axillary lymphnode 2. Nodular lesion chest wall	I: Bottle marked right axillary lymphnode: Received single pale brown nodular tissue measuring 4 × 3 × 2.5 cm^3^, cut surface shows greyish white soft to firm lobulated areas. Also received in same container pale brown nodule measuring 1 × 0.8 × 0.7 cm^3^, cut surface is pale brown. A1 to A3: Section from large nodule‐ 3 bits. B: Section from smaller nodule‐2 all II: Bottle marked nodular lesion chest wall: Received two irregular cut skin covered tissue measuring ‐ 0.8 × 0.7 × 0.6 cm^3^ and 1.8 × 1.4 × 1 cm^3^, the skin surface of large fragment shows focal greyish white areas measuring 0.7 cm across which is reaching upto skin margin. Cut surface of both the fragments shows soft to firm greyish white areas. C1 & C2: Larger skin covered tissue ‐4wall D:Smaller skin covered tissue ‐3all	A1 to A3: Sections show part of the lymph nodal tissue showing effaced architecture with bands of fibrous septate showing atypical lymphoid infiltrates with eosinophilia and patchy necrosis and xanthogranulomatous inflammation. B: Section shows part of lymph node showing non‐specific reactive changes. C1, C2 &D: Sections show skin overlying dermis showing multiple nodular lesion with similar atypical lymphoid infiltrates associated with brisk inflammation, xanthogranulomatous reaction and necrosis.	Right axillary lymph node and “nodular lesion,” chest wall, excision biopsy‐ atypical lymphoid infiltrates, consistent with lymphoma	The possibilities include Hodgkin's lymphoma /anaplastic large cell lymphoma (ALCL). Immunohistochemistry is mandatory for confirmation and for further subcategorization.

**TABLE 5:** Immunohistochemistry of specimen collected from the patient before chemotherapy

Specimen collection date	Specimen	Test	Microscopic lesions	Impression
March 20, 2019	Right axillary lymph node and “nodular lesion,” chest wall, excision biopsy	Lymphoma panel	CD3 ‐ Negative CD20 ‐ Negative CD15 ‐ Positive(FEW, *LARGE* CELLS) CD30 ‐ Positive (Large atypical cells) LCA ‐ Negative (Large atypical cells) LMP ‐ Negative PAX5 ‐ Positive(Weak, Large atypical cells) ALK‐1 ‐ Negative OCT‐2 ‐ Negative BOB‐1‐ Negative CD56‐ Negative ERBER‐ISH ‐Positive (Few cells)	Right axillary lymph node and nodular lesion, chest wall, excision biopsy: consistent with classical Hodgkin lymphoma, nodular sclerosis.

### 2.6 Treatment

There is no specific treatment for HL with skin lesions. Standard treatment is chemotherapy with ABVD/ BEACOPP/Stanford V with or without involved field radiation depending on the stage and bulk of the disease.^1^, ^8^ Among these, the most common chemotherapy protocol used as in our present case is ABVD (*doxorubicin*, *bleomycin*, *vinblastine*, *dacarbazine*), that is, doxorubicin 25 mg/m^2^, bleomycin 10 mg/m^2^, vinblastine 6 mg/m^2^, and dacarbazine 375 mg/m^2^ as days 1 and 15 schedules and to be repeated after every 28 days for six cycles^1^ (Table 6).

**TABLE 6:** List of chemotherapy agents utilized in the case

Regimen	Description	Number of cycles given	Reference
ABVD	Inj. DOXORUBICIN 40 mg in 100 ml NS over 30 min Inj. BLEOMYCIN 16 U dissolve in 5 ml NS and administer as a show iv push over 10 min Inj. VINBLASTINE 9.6 mg iv push in running saline Inj. DACARBAZINE 600 mg in 500 ml 5% dextrose over 2 h (use separate line)	6	1, 8

### 2.7 Outcome and follow‐up

**FIGURE 3:**
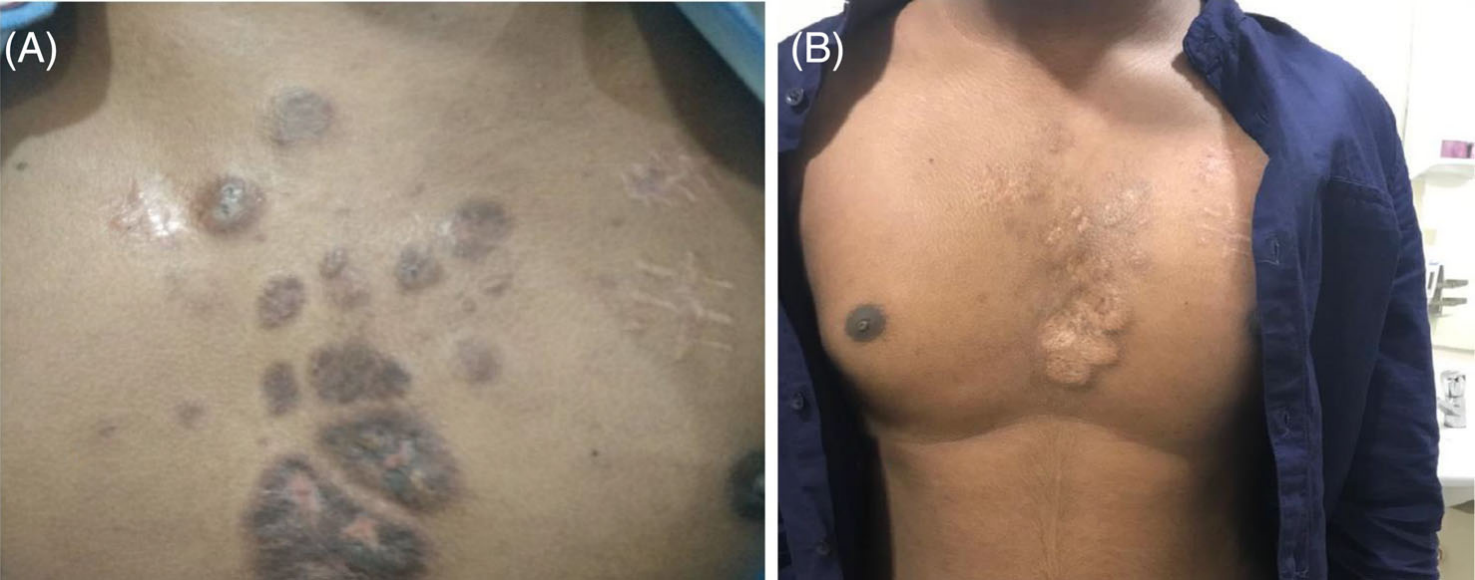
Post‐chemotherapy photograph showing mid‐chest region with good remission. (A) After 3 cycle of chemotherapy. (B) After 6 cycle of chemotherapy

**FIGURE 4:**
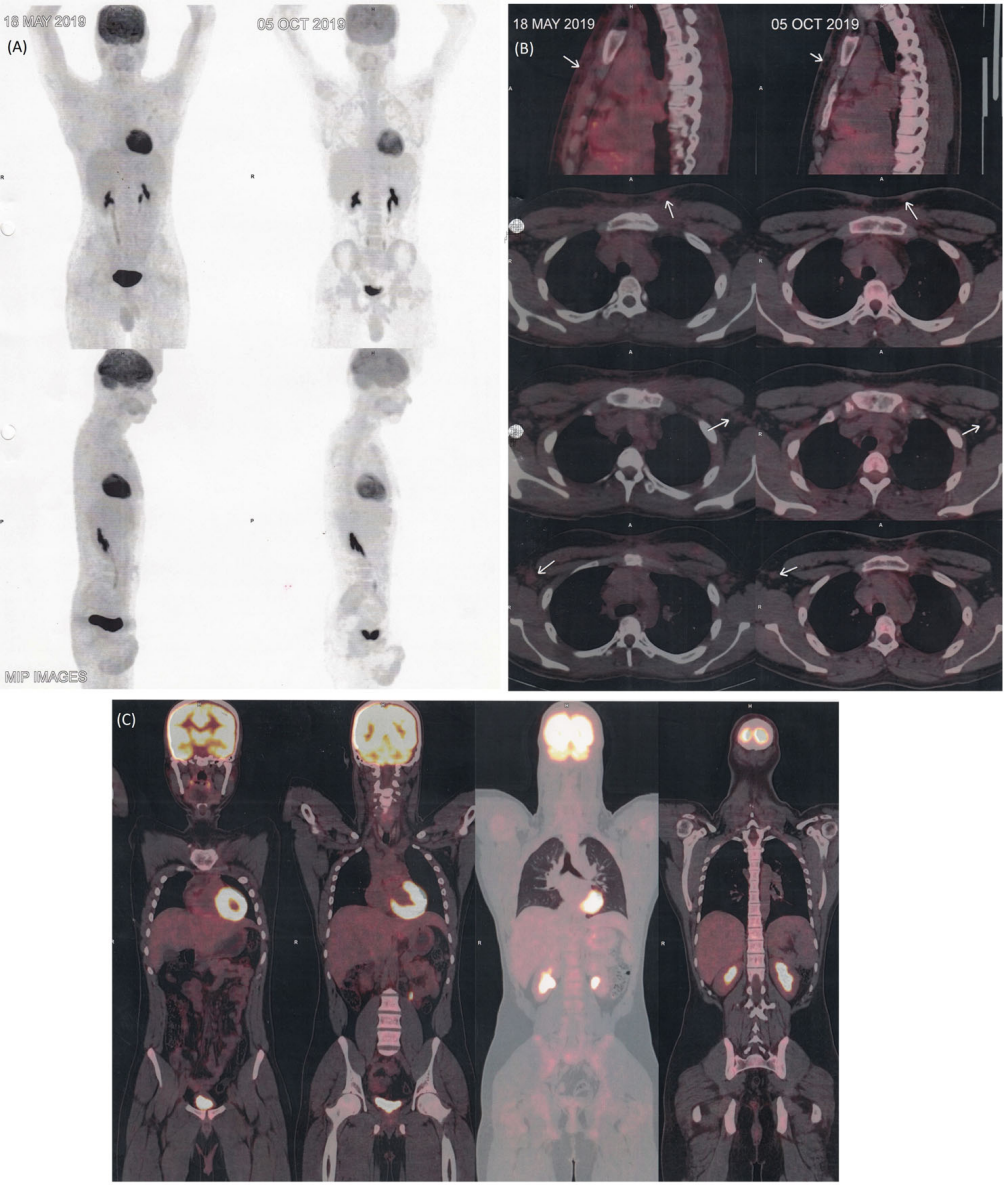
Comparative positron emission tomography‐computed tomography (PET‐CT) and CT imaging during 3 cycle of ABVD and 1 month after 6 cycle of ABVD. (A) A whole body scan showing regression of the primary pathology involving the lymph node (left) and PET scan done after 6 cycle of ABVD showing regression in size with resolution of metabolic activity of supraclavicular, axillary and mediastinal nodes (right). (B–C) There is further regression in size of the supraclavicular, bilateral axillary, subpectoral, deep pectoral, bilateral internal mammary, and perivascular nodes are seen

Overall, HL has a good prognosis with greater than 80% 5‐year survival.^8^ However, cutaneous involvement is usually associated with diffuse lymphadenopathy, late‐stage disease, and poor prognosis.^1^ In our case, the outcome was favourable with a good dermatological response. To date, the patient has completed the six planned cycles, showing significant clinical improvement in both lymphadenomegalies and in skin lesions with no sign of swelling chest wall (Figures 3 and 4) (Tables S1–S4). Imaging features are suggestive of significant response to therapy with residual disease. He is doing well 18 months after stopping chemotherapy, with no evidence of lymphoma recurrence.

## 3 DISCUSSION

HL is responsible for 0.58% of all diagnosed cancers, where a slight male predominance exists (1.1:1). HL incidence has a bimodal peak depending on age, early peak, from 25 to 30 years of age, and a second peak, from 75 to 80 years of age.^1^ The aetiology of this disease and its pathogenesis remain unknown. However, a few risk factors have been involved, such as genetic predisposition, Epstein‐Barr virus or HIV infections, and immune suppression.^4^ World Health Organization (WHO) classified this disease into two main groups: the rare lymphocyte‐predominant nodular and classical HL. There are four subtypes of classical HL: nodular sclerosis, mixed cellularity, lymphocyte‐rich, and lymphocyte‐depleted. Each sub‐type has its clinical characteristics, pathological features, and prognosis.^9^ HL patients typically undergo painless lymphadenopathy. One‐third of patients have B symptoms, such as unexplained fever, drenching night sweats, weight loss, and poor prognosis.^9^ About 90% of patients with HL have contiguous sites of involvement. Visceral involvement with HL can be secondary to the spread from adjacent regions of the lymph node or maybe haematogenous, such as nodular disease in the liver, spleen, or multiple bone locations.^8^, ^9^ HL is rarely associated with intestinal lymphoid tissues such as Waldeyer's ring and Peyer's patches. Often, the upper aerodigestive tract, central nervous system, and skin are only rarely involved.^4^ In some clinical observations, the frequency of cutaneous lesions associated with HL infiltration was recorded as 0.5%–3.4%; lesions are frequently associated with advanced‐stage disease, and the most common findings are plaques, nodules, papules, and ulcers.^10^ Various tumour dissemination mechanisms may explain skin involvement: retrograde lymphatic diffusion from pathological lymph nodes is thought to be the most common. Two other pathophysiological patterns are the direct extension due to contiguity and haematogenous spread.^8^ Differential diagnosis must be made between HL skin involvement and other histological subtypes, such as fungal mycosis, lymphomatoid papulosis, anaplastic large cell lymphoma, and granulomatous slack skin disease.^11^

The extent of intrathoracic spread can be measured by CT scanning of the chest. An abdominal and pelvic CT scan should be performed to assess enlarged nodes in the retroperitoneal area, hepatosplenomegaly, or focal nodules in the spleen or liver.^4^ For initial staging and a follow‐up study to assess residual disease, FDG‐PET is more sensitive than CT. A needle biopsy of the posterior iliac crest bone marrow should be restricted to patients with B symptoms or clinical evidence of subdiaphragmatic disease, as the yield is meagre in asymptomatic patients with limited clinical disease. The overall incidence of bone marrow involvement in HL is just 5%. As indicated by the Ann Arbor staging system, skin involvement in HL is stage IV disease.^4^ Our case presented multiple cutaneous nodular lesions over the anterior chest wall's skin, and enlarged lymphadenopathy in the supradiaphragmatic regions with a bottle marked right axillary lymph node. In resource‐limited settings, it is very difficult to diagnose and manage such cases. When the patient first presented with nodules and B symptoms, he was taken to the local hospital and treated with broad‐spectrum antibiotics prescribed by the general practitioner showing no signs of improvement. After that he was taken to a district level hospital where he was given anti‐tubercular treatment for approximately 2 months but failed to find any relief. The success of biopsies is closely related to the choice of indications. Unfortunately, both physicians at local and district level did not suggest for biopsy which hindered the diagnostic biopsy. Atypical lymphoid infiltrates, consistent with lymphoma in histopathology and the classical immunoexpression pattern, showed positive CD15, CD30, PAX5. There is no specific treatment for cutaneous HL localisation: six ABVD cycles are recommended for advanced‐stage disease. However, this schedule must be validated by achieving an early metabolic response reported by a PET/CT scan after the first two cycles of chemotherapy.^12^ The patient has received six cycles of chemotherapy, with good tolerance to treatment, and responds significantly both in the adenopathies and in the cutaneous localisations. With the advent of novel agents, brentuximab‐vedotin and immune checkpoint inhibitors have a significantly role to improve the treatment outcome, even previously failed to multiple lines of chemotherapy.^13^

While cutaneous Hodgkin lymphoma is fairly rare, it should be considered among the atypical causes of non‐specific papules, plaques, and ulcers, necessitating the consultation of a hematopathologist. In contrast to the majority of recorded cases of HL affecting the skin, our case shows that a successful response can be achieved with appropriate therapy.

## CONFLICT OF INTEREST

The authors declare no conflicts of interest.

## AUTHORS' CONTRIBUTIONS

All authors had full access to the data in the study and take responsibility for the integrity of the data and the accuracy of the data analysis. *Conceptualization*, Su.G., Sa.G., N.S.P., S.C.; *Data Curation*, Su.G., Sa.G.; *Validation*, Sa.G., R.J.A., F.C.; *Writing ‐ Original Draft*, Su.G.; *Writing ‐ Review & Editing*, Sa.G., R.J.A., F.C., N.S.P., P.P., S.C.; *Visualization*, S.C.; *Supervision*, P.P., S.C.

## ETHICAL STATEMENT

The authors certify that they have obtained all appropriate patient consent forms. In the form, the patient has given his consent for his images and other clinical information to be reported in the journal. The patients understand that their names and initials will not be published, and due efforts will be made to conceal their identity.

## Supporting information


**Data S1.** Supporting information.

## Data Availability

The data that support the findings of this study are available from the corresponding author upon request.
