# Supplementary material for: Classical Hodgkin's lymphoma with cutaneous involvement in an adolescent male: A case study
Source: Cancer Rep (Hoboken). 2021 Jun 5;5(2):e1473. doi: 10.1002/cnr2.1473 (PMC8842694; doi:10.1002/cnr2.1473)
Supplement: Supplementary file 1 — Data S1. Supporting information. [file CNR2-5-e1473-s001.pdf]

Classical Hodgkin's Lymphoma with cutaneous involvement in an adolescent male: a case study

Sumon Ghosh[a*], Sajib Ghosh[b], Rownak Jahan Amin[c], Fahmida Chowdhury[a], Namala Satya Prasad[d], Pandurangan Prabu[d], Sukanta Chowdhury[a]

[a] International Centre for Diarrhoeal Disease Research, Bangladesh (icddr,b)

[b] Eastern Medical College & Hospital, Cumilla, Bangladesh

[c] National Institute of Cancer Research and Hospital, Dhaka, Bangladesh

[d] Apollo Hospital, Greams Road, Chennai, India

*Correspondence: icddr,b, Mohakhali, Dhaka 1212, Bangladesh.
E-mail*:* sumon.ghoshbd@gmail.com (S. Ghosh)

Supplementary documents:

Radiological examination:

PET-CT (Positron Emission Tomography–Computed tomography) whole body:

CT Report: 25.03.2019

CLINICAL HISTORY :

Anterior chest wall skin lesion (HPE: fungal infection), axillary lymphadenopathy (Biopsy – lymphoma),for evaluation

CT FINDINGS

Multislice (64 slice) serial axial section of head and mid thigh were studied without administration f IV and oral contrast.

Brain

Cerebral neuroparenchyma shows normal attenuation, enhancement and gray white matter differentiation. Brain stem and cerebellum are normal. Ventricles and cisterns are normal. Calvarium shows no destructive lesion.

Head and Neck

Prominent bilateral level II and V nodes are noted.

Base of skull, orbits, paranasal sinuses, naso-, oro- and hypopharynx and larynx are normal .

Bilateral carotid arteries and jugular veins are normal.

The parotid and submandibular glands are normal. Thyroid glands are normal.

Chest

Multiple enlarged left supraclavicular , bilateral axillary, subpectoral ,deep pectoral, bilateral internal mammary and prevascular nodes are seen, largest left internal mammary nodal mass extending into the left chest wall measures 6.0 x 5.5cms and left axillary node measures 3.2 x3.0 cms.

Multiple ill defined soft tissue are seen in the anterior chest wall muscles. Areas of necrosis are noted within these deposits.

Multiple well defined cutaneous soft tissue deposits are also seen in the chest wall, largest measures 4.0 x2.5cms. Ulceration IS noted in few of the lesions.

Nodular pleural thickening is also seen in the bilateral upper hemithorax. No evidence of pleural effusion.

Lungs show no suspicious mass or nodules .

The cardia, pulmonary trunk and aorta are normal. No pericardial effusion.

Abdomen and Pelvis

Liver is normal size. No focal lesion is seen her liver. No intra hepatic biliary radicle dilatation. Portal vein, hepatic veins and IVC ARE normal.

Gall bladder, spleen, pancreas, adrenals and both kidneys are normal.

No significant paraaorticadenopathy .No mesenteric or peritoneal deposits . No ascites

The urinary bladder, prostate and seminal are normal.

No significant pelvic are adenopathy

PET REPORT:

Fusion PET/CT imaging was performed from the vertex of skull to mid thigh 60 mins after IV administration of 4.3 mCi of F-18 fluorodeoxyglucose (FDG). CT was performed for the purpose of attenuation correction and anatomical correction. Blood glucose level was 124 mg/dl prior to scan.

Head & Neck

Increased FDG uptake is noted in the enlarged left supraclavicular node (SUV max 10.4). Physiological FDG uptake is noted in the rest of head and neck. No significant FDG avid cervical nodes are seen.

Thorax

Increased FDG uptake is noted in the enlarged axillary / subpectoral (SUV max right 8.8; left 10.6), prevascular (SUV max 8.6), paraaortic (SUV max 7.1) anterior cardiophrenic (SUV max 9.1) and supradiaphragmatic (SUV max 8.1) nodes.

Increased FDG uptake is noted in the large pleural based deposit in the left internal mammary region (SUV MAX 11.5).

Increased FDG uptake is noted in the pleural based deposits in both hemithorax (SUV max 9.3)

Increased FDG uptake is noted in the deposits noted is bilateral pectoralis muscle (SUV max right 10.4; left 7.7)

Increased FDG uptake is noted in the cutaneous and subcutaneous deposits in anterior chest wall (SUV max 9.3)

No significant FDG uptake is noted in fissural nodule in left hemithorax

No abnormal FDG uptake is noted in the rest of lungs.

Abdomen & Pelvis

No abnormal FDG uptake is seen in the liver, spleen, gallbladder, pancreas, kidneys and adrenals.

FDG distribution in the bowel loops is in a physiological pattern.

No significant FDG avid abdominal, pelvic or inguinal nodes are seen.

Bone

No demonstrable abnormal FDG uptake noted in the bones and bone marrow.

PET –CT IMPRESSION

Anterior chest wall skin lesions (HPE –fungal infection),axillary lymphadenopathy (Biopsy- Lymphoma), for evaluation

1) **Hypermetabolic cutaneous and subcutaneous lesions in anterior chest wall.**
2) **Hypermetabolic supraclavicular axillary and mediastinal nodes**
3) **Hypermetabolic anterior chest wall muscle deposits.**
4) **Hypermetabolic pleural deposits in bilateral hemithorax.**
5) **No other demonstrable metabolically active disease elsewhere in the whole body survey.**

Rx: Chemotherapy

6 cycle/12 dose

Dose no: 1 Day 1 **Date**: 27.03.2019

Ht: 167 cm Wt: 56.2 Kg BSA: 1.6 M^2

Premedication

Inj. Emeset 8mg IV stat

Inj.Dexamethasone 8mg in 1ooml NS

NS 500ml iv over 1 hour

Inj. DOXORUBICIN 40mg in 100ml NS over 30minutes

Inj. BLEOMYCIN16 U dissolve in 5 ml NS and administer as a show iv push over 10 minutes

Inj. VINBLASTINE 9.6 mg iv push in running saline

Inj. DACARBAZINE 600mg in 500ml 5% Dextrose over 2 hour(use separate line)

Oral medication

1.Tab.Pan 40 PO 1-0-0(before breakfast)*5 days

2.Tab. Emeset 4mg PO1-1-1 (after meals)*3 days

3.Tab.Febutaz 40 mg PO 1-0-0(after meals)*5 days

After Chemotherapy

After first dose of Chemotherapy

Table S1: Biochemical test

**Specimen collection date**	**Specimen**	**Test name**	**Result**	**Biological reference intervals**	**Units**
28.03.2019	Serum	Urea - Serum/Plasma(UREASE-GLDH-UV)<br>Creatinine – Serum / Plasma (Jaffe Kinetic)<br>Potassium- Serum (Ion-Selective Electrode: ISE Technology)	26<br>0.6<br>4.7	11-39<br>0.5-1.0<br>3.4-4.7	mg/dL<br>mg/dL<br>mg/dL
01.04.2019	Whole Blood (EDTA)	**CBC**<br>Hemoglobin(Modified Cyanmethhaemoglobin)<br>Packed cell volume (Calculated)	 10.8*<br>35*	 13-16<br>37-49	 mg/dL<br>%

		WBC Count (Optical(Light scatter) & Microscopy)	9.18	4.5-13.5	$10^3/mm^3$
		Platelet Count (Optical (Light scatter))	527*	150-450	$10^3/mm^3$
		ESR (Automated – Westergren method)	24*	0-15	Mm/hr
		**Differential Count (Optical(light scatter)/VCS/Microscopy)**			
		Neutrophils			
		Lymphocytes	58	33-76	%
		Eosinophils	39	15-55	%
		Monocytes	2	0-3	%
			1	0-4	%
08.04.2019	Serum	Urea-Serum/Plasma (Urease-GLDH-UV)	17	11-39	mg/dL
		Creatinine-Serum/Plasma (Jaffe Kinetic)	0.8	0.5-1.0	mg/dL
		Potassium-Serum (Ion-Selective Electrode: ISE Technology)	4.3	3.4-4.7	mEq/L
		**Liver Function Test** (Package)			
		Bilirubin, Total- Serum (Vanadate Oxidation-endpoint)	0.3	0.0-1.3	mg/dL
		Bilirubin, Conjugated (Direct)- Serum (Vanadate Oxidation-endpoint)	0.1	0.0-0.4	mg/dL
		Bilirubin, Unconjugated- Serum (Calculated)			
		**Protein Total**- Serum/Plasma (Biuret)	0.2	0.0-1.2	mg/dL
		Albumin-Serum	8.3*	6.0-8.0	g/dL
		Globulin-Serum: (Calculated)	4.4	14-18 Years: 3.2-	g/dL
		AST (SGOT)-Serum (IFCC)	3.9*	4.5	g/dL
		ALT (SGPT)-Serum/Plasma (IFCC)	28	2.0-3.5	U/L
		GGTP: Gamma GlutamylTranspeptidase-Serum	31	15-45	U/L
		Alkaline Phosphatase – Serum/Plasma (IFCC Modified AMP buffer)	44	1-19 Years: 5-45	U/L
				Male: <55	
			126		U/L
				<260	

After 4 dose of chemotherapy

Table S2: Biochemical test

**Specimen collection date**	**Specimen**	**Test name**	**Result**	**Biological reference intervals**	**Units**
17.05.2019	Whole blood (EDTA)	**Hemogram**(Automation)			
		Hemoglobin(Modified Cyanmethhaemoglobin)	12.8*	13-16	gm%
		Packed cell volume (Calculated)	39		
		WBC Count (Optical (light scatter) & Microscopy)	5.52	37-49	%
		Platelet Count (Optical (light scatter))	377	4.5-13.5	$10^3/mm^3$
		ESR (Automated – Westergren method)	6	150-450	$10^3/mm^3$
		RDW (Derived from RBC histogram)	20*	0-15	mm/hr
"	"	**Differential Count (Optical(light scatter)/VCS/Microscopy)**			%
		Neutrophils	47	33-76	%
		Lymphocytes	42	15-55	%
		Eosinophils	6*	0-3	%
		Monocytes	5*	0-4	
		**Microscopy**			
		RBC	Normocytic hypochromic RBCs		
		WBC	Differential within normal limits		
		Platelets	Adequate on the smear		
01.04.2019	Whole Blood	**CBC**			

	(EDTA)	Hemoglobin(Modified Cyanmethhaemoglobin)	13.6	13-16	mg/dL
		Packed cell volume (Calculated)	42	37-49	%
		WBC Count (Optical(Light scatter) & Microscopy)	6.73	4.5-13.5	$10^3/mm^3$
		Platelet Count (Optical (Light scatter))	387	150-450	$10^3/mm^3$
		ESR (Automated – Westergren method)	5	0-15	Mm/hr
		**Differential Count (Optical(light scatter)/VCS/Microscopy)**			
		Neutrophils		33-76	%
		Lymphocytes	58	15-55	%
		Eosinophils	33	0-3	%
		Monocytes	3	0-4	%
			6*		

Radiological examination:

Date: 18.May.2019

PET-CT whole body:

CT Report:

CLINICAL HISTORY :

Classical Hodgkin’s lymphoma, post 2 cycles of chemotherapy (last dose on 8 -5-2019) , deep fungal infection in anterior chest wall, on antifungal, for response evaluation.

CT FINDINGS

Multislice (64 slice) serial axial section of head and mid thigh were studied without administration of IV and oral contrast

Brain

Cerebral neuroparenchyma shows normal attenuation, enhancement and gray white matter differentiation. Brain stem and cerebellum are normal. Ventricles and cisterns are normal. Calvarium shows no destructive lesion.

Head and Neck.

Base of skull, orbits, paranasal sinuses, naso-, oro- and hypopharynx and larynx are normal .

Bilateral carotid arteries and jugular veins are normal .Prominent left supraclavicular lymph node measuring 0.9 x 0.4

The parotid and submandibular glands are normal. Thyroid glands are normal.

Chest

Significant regression in the previously seen enlarged left supraclavicular , bilateral axillary, subpectoral ,deep pectoral, bilateral internal mammary and pervascular nodes are seen, largest left internal mammary nodal mass extending into the left chest wall measures 1.5x1.5 cms, previously measured 6.0x 5.5 cms and left axillary node measures 1.3x1.2cms, previously measured 3.2x3.0 cms.

Significant regression in the multiple ill-defined soft tissue and subcutaneous, cutaneous deposits are seen in the anterior chest wall muscles. Larger cutaneous soft tissue deposits are also seen in the chest wall, largest measures 4.0 x 2.5 cms.

Resolution of the nodular pleural thickening is also seen in the bilateral upper hemithorax. No evidence of pleural effusion.

Lungs show no suspicious mass or nodules.

The cardia, pulmonary trunk and aorta are normal. No pericardial effusion.

Abdomen and Pelvis

Liver is normal in size. No focal lesion is seen her liver. No intra hepatic biliary radicle dilatation. Portal vein, hepatic veins and IVC are normal.

Gall bladder, spleen, pancreas, adrenals and both kidneys are normal.

No significant paraaorticadenopathy.No mesenteric or peritoneal deposits . No ascites.

The urinary bladder, prostate and seminal vesicles are normal.

No significant pelvicadenopathy.

PET REPORT:

Fusion PET/CT imaging was performed from the vertex of skull to mid thigh 60 mins after IV administration of 4.9mCi of F-18 fluorodeoxyglucose (FDG). CT was performed for the purpose of attenuation correction and anatomical correction. Blood glucose level was 92 mg/dl prior to scan.

Head & Neck

No significant FDG uptake is seen in the prominent left supraclavicular node [Deauville's score l].

Non FDG avid bilateral level ll cervical nodes are seen-likely reactive

Physiological FDG uptake is noted in the rest of head and neck.

Thorax

Mildly increased FDG uptake is noted in the prominent bilateral axillary (SUV max 2.5), subpectoral, paraaortic, anterior cardiophrenicand supradiaphragmatic nodes [Deauville's score lll].

Low grade FDG uptake is noted in the deposits noted in bilateral pectoral muscle (SUV max2.6 on left) and cutaneous, subcutaneous deposits in anterior chest wall (SUV max 2.6).

No significant FDG uptake is noted in fissural nodule in left hemithorax.

No abnormal FDG uptake is noted in the rest of lungs.

Abdomen & Pelvis

No abnormal FDG uptake is seen in the liver, spleen, gallbladder, pancreas, kidneys and adrenals.

FDG distribution in the bowel loops is in a physiological pattern.

No significant FDG avid abdominal, pelvic or inguinal nodes are seen.

Bone

No demonstrable abnormal FDG uptake noted in the bones and bone marrow.

PET –CT IMPRESSION

Classical Hodgkin's lymphoma, post 2 cycles of chemotherapy (last dose on 08-05-2019), deep fungal infection in anterior chest wall, on antifungals, for response evaluation.

In comparison with previous PET/CT scan performed on 25-03-2019, there is interval

1) **Significant regression in number, size and metabolic activity of cutaneous lesions and intramuscular deposits on anterior chest wall.**
2) **Significant regression in size and metabolic activity of axillary and mediastinal nodes with resolution of metabolic activity of supraclavicular nodes.**
3) **Resolution of bilateral plural deposits.**
4) **No new lesion.**

AFTER 6 CYCLES OF CHEMOTHERAPY
Table S3: Biochemical test (Haematology)

**Specimen collection date**	**Specimen**	**Test**	**Result**	**Biological reference intervals**	**Units**
**02.10.2019**	**Whole blood (EDTA)**	**Peripheral blood smear(PBS):(microscopy)**<br>**Microscopy**<br>**RBC**<br><br>**WBC**	 <br>Normocytic normochromic cells<br>Mild		

		**Platetets**<br><br>**RENAL PACKED -1**<br>**HEMOGLOBIN (AUTOMATION)**<br><br>Hemoglobin(Modified<br>Cyanmethaemoglobin)<br>**PACKED CELL VOLUME**<br>**[PCV]-(Automation):** (calculated)<br>**TOTAL LEUCOCYTE**<br>**COUNT(TLC):(**OPTICAL/impedance)<br>WBC COUNT (optical(light scatter)<br>&Microscopy)<br>**Platelet count**<br>**:(OPTICAL/impedance)**<br>Platelet count (optical(light<br>scatter)<br>**Differential Leucocytic count**<br>(OPTICAL/impedance/microscopy)<br>Neutrophils<br>Lymphocytes<br>Eosinophils<br>Monocytes<br><br>**Comments :** NOTE : Kindly<br>correlate values with clinical<br>finding	eosinophilia<br>noted<br>Adequate on<br>the smear<br><br><br><br><br>14.9<br><br>47<br><br><br>8.4<br><br><br><br>290<br><br><br><br>53<br>35<br>7*<br>5*	**13 – 16**<br><br>**37 – 49**<br><br><br>**4.5 -13.5**<br><br><br><br>**150 – 450**<br><br><br><br>**33 – 76**<br>**15 – 55**<br>**0 – 3**<br>**0 – 4**	**gm/dl**<br><br>**%**<br><br><br>**$10^3/mm^3$**<br><br><br><br>**$10^3/mm^3$**<br><br><br><br>**%**<br>**%**<br>**%**<br>**%**

CT REPORT

CLINICAL HISTORY

Classical **Hodgkin's** lymphoma, deep fungal infection in anterior chest wall, on antifungal, post 6 cycles of chemotherapy (last dose on 28 - 08-2019), for evaluation

CT FINDINGS

Multislice (64 slice) serial axial section of head to mid thigh were studied withput administration of IV and oral contrast

Brain

Cerebral neuroparenchyma shows normal attenuation, enhancement and gray white matter differentiation. Brain stem and cerebellum are normal. Ventricles and cisterns are normal. Calvarium shows no destructive lesion

HEAD AND NECK

No significant cervical adenopathy .

Base of skull, orbits, paranasal sinuses, naso-, oro- and hypopharynx and larynx are normal .

Bilateral carotid arteries and jugular veins are normal .

The parotid and submandibular glands are normal. Thyroid glands are normal.

CHEST

There is further regression in size of the supraclavicular, bilateral axillary, subpectoral ,deep pectoral, bilateral internal mammary and pervascular nodes are seen, largest left internal mammary nodal mass extending into the left chest wall measures 1.2x1.0cms, previously measured 1.5x 1.5cms and left axillary node measures 1.0x0.8cms, previously measured 1.3x1.2cms,Stable multiple ill definedsoft tissue and subcutaneous, cutaneous deposits are seen in the anterior chest wall, larger cutaneous soft tissue deposits measures 4.0 x1.2cms,previously measured 4.0x 2.5cms

No pleural effusion.

Abdomen and pelvis

Liver measures 15.5cms. No focal lesion is seen in the liver. No intra hepatic biliary radicle dilatation. Portal veinsand IVC are normal.

Gall bladder, spleen, pancreas, adrenals and both kidney are normal.

No significant paraaorticadenopathy. No mesenteric or peritoneal deposits. No ascites.

The urinary bladder, prostate and seminal vesicles are normal.

No significant pelvic adenopathy.

PET REPORT

Fusion PET/CT imaging was performed from the vertex of skull to mid thigh 60 mins after IV administration of 7.0mCi of F-18 fluorodeoxyglucose (FDG). CT was performed for the purpose of attenuation correction and anatomical correction. Blood glucose level was 105 mg/dl prior to scan.

HEAD & NECK

No abnormal FDG avid supraclavicular nodes are seen

Non FDG avid bilateral level ll cervical nodes are seen-likely reactive

Physiological FDG uptake is noted in the rest of head and neck.

Thorax

No significant FDG uptake is seen in the bilateral axillary, subpectoral, internal mammary and paraaortic nodes [Deauville's scope 1]

Supra Prominent anterior cardiophrenic and supradiaphragmatic node have resolved.

No abnormal focal FDG uptake is seen in the pectoral muscles.

No significant FDG uotake is seen in the cutaneous and subcutaneous deposits in the anterior chest wall.

No significant FDG uptake is noted is fissural nodule in left hemithorax.

No abnormal FDG uptake is noted in the rest lungs.

Abdomen and pelvis

Tracer stasis is noted in the bilateral pelvicalyceal system and ureters

No abnormal FDG uptake is seen in the liver, spleen, gallbladder, pancreas, kidneys and adrenals

FDG distribution in the bowel loops is in a physiological pattern

No significant FDG avid abdominal, pelvic or inguinal nodes are seen.

Bone

No demonstrable abnormal FDG uptake noted in the bones and bone marrow

PET – CT IMPRESSION

Classical Hodgkin's lymphoma, deep fungal infection in anterior chest wall, on antifungal, post 6 cycles of chemotherapy (last dose 28.08.2019), for evaluation.

In comparison with previous PET/CT scan performed on 18-05-19, there is interval

1. **Resolution of metabolic activity of cutaneous lesions in anterior chest wall while the intramuscular deposits have resolved.**
2. **Resolution of anterior cardiophrenic and supradiaphragmatic nodes**
3. **Regression in size with resolution of metabolic activity of supraclavicular, axillary and mediastinal nodes**
4. **No new lesion**

Imaging features are suggestive of complete metabolic response

Table S4: Biochemical test (Haematology)

**Specimen collection date**	**Specimen**	**Test name**	**Result**	**Biological reference intervals**	**Units**
02-10-	Serum	**LDH:LACTATE DEHYDROGENASE –SERUM**	**252**	>12 years:125-	U/L

2019		**(**LACTATE/NAD –COLORIMETRY,AUTOMATED)		220	
		**RENAL PACKAGE –l**<br>**GLUCOSE –PLASMA (RANDOM) (GLUCOSE Oxidase Trinder –endpoint)**	**102**	<140	mg/dl
		**Urea –SERUM/PLASMA**<br>(UREASE-GLDH-UV)	**26**	**11-39**	mg/dl
		**CREATININE- SERUM/PLASMA**<br>(Jaffe, Alkaline picrate, kinetic –IDMS standardization)	**1.0**	**0.5-1.0**	mg/dl
		**Uric acid –serum**<br>(URICASE/PEROXIDASE)	**10.2**	**Male:3.5-7.2**	mg /dl
		**CHOLESTEROL-SERUM/PLASMA**<br>(Enzymatic method)	**150**	Desirable :<170<br>Borderline High:170-199<br>High :>=200	mg /dl
		**BILIRUBIN, TOTAL –SERUM**<br>**VANADATE Oxidation-endpoint :**	**0.7**	0.0-1.3	mg/dl
		**BILIRUBIN CONJUGATED (DIRECT)-SERUM**<br>**(Vanadate oxidation)-endpoint**	**0.2**	0.0-0.4	mg/dl
		**BILIRUBIN UN CONJUGATED –SERUM(Calculated)**<br>**(This parameter is not in the scope of NABAL accreditation)**	**0.5**	0.0-1.2	mg/dl
		**PROTEIN TOTAL –SERUM /PLASMA**<br>(Biuret)	**7.7**	6.8-8.0	g/dl
		**ALBUMIN- SERUM**<br>(BCG)	**4.9**	14-18 Years: 3.2-4.5	g/dl
		**GLOBIN –SERUM :(CALCULATED)**<br>**(This parameter is not in the scope of NABAL accreditation**	**2.8**	2.0-3.5	g/dl
		**ALT(SGPT)-SERUM/ PLASMA**<br>(UV without P5P)	**59**	1-19 Years :5-45	U/L

		**ALKAKINE PHOSPHATASE –SERUM /PLASMA**<br>(PNPP,AMP buffer –kinetic)	**133**	<260	U/L
		**CALCIUM –SERUM**<br>(Arsenoazolll)	**9.7**	8.6-10.2	mg /dl
		**PHOSPHORUS ,INORGANIC –SERUM**<br>**(**phosphomolybdate, UV)	**3.9**	12 -15 Years :2.9-5.4	mg /dl
		**SODIUM –SERUM**<br>(Ion –selective Electrode :Indirect)	**139**	138-145	mEq /L
		**POTASSIUM- SERUM**<br>(Ion –selective Electrode :Indirect)	**4.3**	3.4-4.7	mEq /L
		**CHLORIDE- SERUM**<br>(Ion –selective Electrode :Indirect)	**106**	98-107	mEq /L
		**CARBON DIOXIDE (CO2),TOTAL-SERUM**<br>**(**ENZYMZTIC METHOD)	**27**	23-29	mEq /L
